# Research on Upper Limb Motion Intention Classification and Rehabilitation Robot Control Based on sEMG

**DOI:** 10.3390/s25041057

**Published:** 2025-02-10

**Authors:** Tao Song, Kunpeng Zhang, Zhe Yan, Yuwen Li, Shuai Guo, Xianhua Li

**Affiliations:** 1 Shanghai Key Laboratory of Intelligent Manufacturing and Robotics, School of Mechatronic Engineering and Automation, Shanghai University, Shanghai 200444, China; zhangkunpeng@shu.edu.cn (K.Z.);; 2 Shanghai Golden Arrow Robot Technology Co., Ltd., 701, Building 3, No. 377 Shanlian Road, Baoshan District, Shanghai 200444, China; 3 National Demonstration Center for Experimental Engineering Training Education, Shanghai University, Shanghai 200444, China; 4 School of Mechatronics Engineering, Anhui University of Science and Technology, Huainan 232001, China

**Keywords:** stroke, surface myoelectricity, upper limb rehabilitation robot, interactive control

## Abstract

sEMG is a non-invasive biomedical engineering technique that can detect and record electrical signals generated by muscles, reflecting both motor intentions and the degree of muscle contraction. This study aims to classify and recognize nine types of upper limb motor intentions based on surface electromyography (sEMG) and apply them to the interactive control of an end-effector rehabilitation robot. The research begins with selecting muscles and data preprocessing, incorporating the generation mechanism of sEMG along with the anatomical and kinesiological principles of upper limb muscles. Next, a musculoskeletal model of the upper limb is established and validated through simulations in OpenSim. To avoid the drawbacks of modeling methods, traditional machine learning and deep learning methods are employed to perform a nine-class classification task on the sEMG data, comparing the classification accuracy of different approaches. Finally, the motor intentions extracted using a multi-stream convolutional neural network (MLCNN) are utilized to control the iReMo^®^ end-effector rehabilitation robot, with the system’s motion smoothness and accuracy evaluated through tests involving different trajectories.

## 1. Introduction

Surface electromyography (sEMG) captures bioelectric signals generated by muscle contractions, reflecting muscle activity [1]. These signals can be processed and translated into control commands for robots, enabling them to respond in real time or perform specific actions based on muscle activity. The control methods for sEMG-based robots can be categorized into three types: fully autonomous continuous control, semi-autonomous assistive control, and discrete control. Fully autonomous control can mimic human movements in real time and has seen significant advancements in numerous studies. For instance, Vogel et al. utilized a Gaussian regression process to decode sEMG signals into directional velocity commands for robotic hands, facilitating control in microgravity environments [2]. Similarly, Hagengruber applied a machine learning approach to convert EMG signals from SMA patients into force and velocity commands for robotic hands [3]. Other strategies, such as Weitschat’s spherical linear interpolation motion planning and joint angle mapping by Artemiadis and Kyriakopoulos, allow robots to accurately mimic human movements with minimal delay [4,5,6,7]. Semi-autonomous assistive control methods typically rely on pre-set motion templates to perform specific actions, simplifying complex task execution. For example, Vogel combined visual perception with these templates to guide a robotic arm in tasks like drinking water and opening doors using sEMG signals, while Shenoy et al. extracted movement direction from sEMG signals to control robotic arm movement using predefined parameters, making it suitable for rehabilitation and daily assistive tasks [8]. In discrete control, these methods primarily depend on simple gesture recognition. Murillo recorded sEMG signals with a Myo armband to identify gestures for controlling a multi-axis robotic arm. Hassan compared various classification algorithms for gesture recognition in a 5-DOF robotic arm [9,10].

Overall, the development of sEMG-controlled robotic technology has matured, with different methods offering varying levels of autonomy, precision, and applicability, showcasing a wide range of promising potential. However, despite significant advancements in sEMG control technology, many rehabilitation robots cannot accurately assess a patient’s muscle condition, making it challenging to identify weak muscles or joint movements. While professional physicians can recognize issues and provide targeted single-joint training recommendations, they face challenges in continuously monitoring each patient’s rehabilitation progress. They cannot promptly correct movement abnormalities caused by trunk compensation. Additionally, end-effector rehabilitation robots are relatively disadvantaged when addressing multi-joint rehabilitation training and trunk compensation issues. In clinical rehabilitation, active patient participation is crucial for training effectiveness; however, standard rehabilitation devices often focus only on completing training plans and achieving goals, leading to less than ideal rehabilitation outcomes.

## 2. Problem Formulation

**Figure 1:**
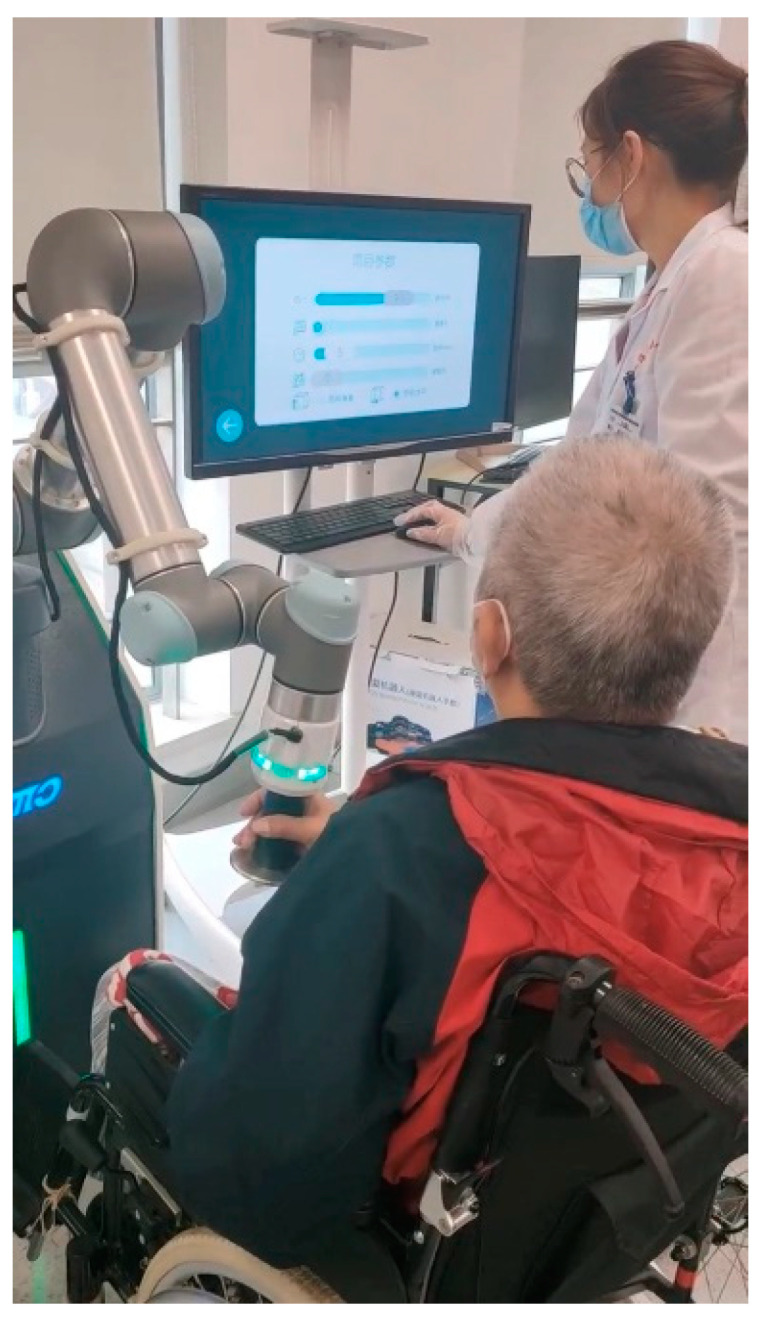
iReMo^®^ upper limb rehabilitation robot system.

**Figure 2:**
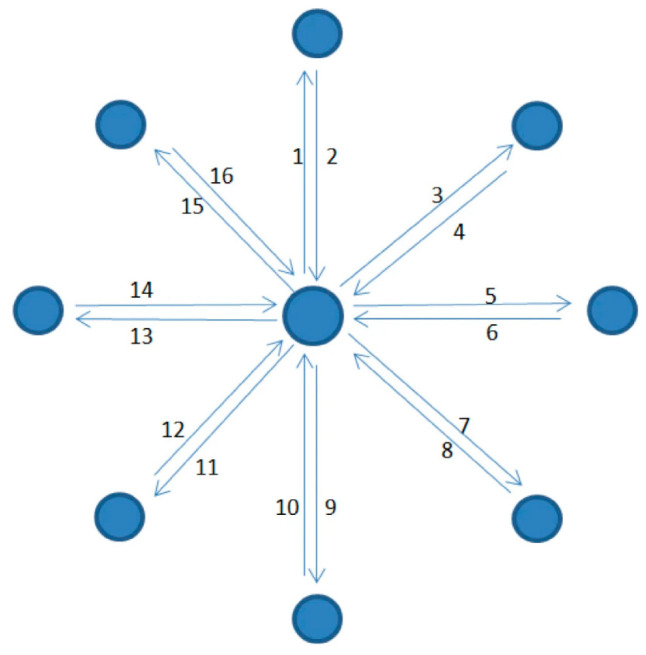
CO-PTP task path.

This study combines surface electromyography (sEMG) technology to improve the deficiencies of the six-degree-of-freedom upper limb rehabilitation robot iReMo^®^ (Shanghai Golden Arrow Robot, Shanghai, China). Currently, upper limb robots are not sufficiently accurate in recognizing the movement intentions of patients. For some patients with weak muscles, the rehabilitation training performed by the robot may be inadequate. Since sEMG signals can be generated before muscle movement, utilizing sEMG can more efficiently and accurately identify the patient’s movement intentions [11]. This approach can provide better patient motion rehabilitation training and more personalized training methods tailored to the individual. Therefore, this paper proposes a control method for driving the robot based on sEMG signals [12], as shown in Figure 1. First, it is necessary to identify the muscles driving upper limb movement and use the sEMG signals generated by these muscles to recognize the patient’s intentions and classify limb movements. As shown in Figure 2, considering the limitations of existing training methods, this study adopts the CO-PTP (center-out point-to-point) task for training. This task helps to improve the range of motion, enhance muscle strength and flexibility, and improve coordination and accuracy of movement [13]. Finally, the classification results will be applied to the control of the robotic arm, thereby enabling rehabilitation training for the patient through the robotic arm.

## 3. Classification of Upper Limb Movements Based on sEMG

### 3.1. Upper Limb Muscle Selection

Computational models are a powerful tool in biomechanics research, and the open-source software OpenSim4.5 provides a modeling platform for human and environmental interactions and is widely used for motion simulation. Holzbaur et al. developed an upper limb model that includes 15 degrees of freedom and 50 muscle compartments to estimate muscle forces and joint moments, aiding the study of neuromuscular control and surgical simulations [14]. Saul et al. constructed the same upper limb model on both OpenSim and SIMM platforms, conducting simulations with electromyographic (EMG) driving, CMC driving, and gravity driving, comparing the simulation results and speed differences for the wrist, elbow, and shoulder joints, thereby validating the performance differences between the two platforms [15]. Liu et al. created a musculoskeletal model driven by EMG signals, providing real-time stiffness control for a bilateral upper limb exoskeleton rehabilitation device by integrating muscle activation with the Hill model [16]. David G. investigated a knee joint musculoskeletal model driven by surface EMG (sEMG) signals, accurately predicting the knee joint moment [17]. Through these representative studies, the application of OpenSim in simulating muscle forces and motion has been extensively validated and developed.

**Figure 3:**
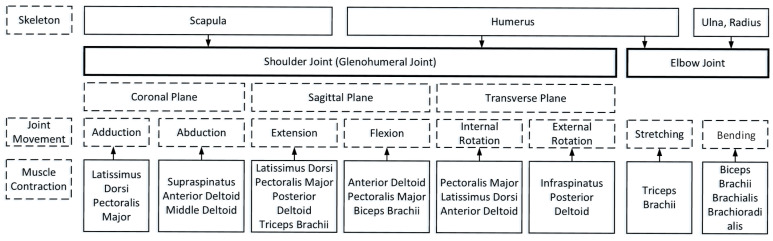
Upper limb shoulder and elbow joint musculoskeletal model content.

The simplified musculoskeletal model (Figure 3) consists of three bones—the scapula, humerus, and forearm—and eleven muscles, simulating shoulder and elbow joint functions. It allows for adduction, abduction, flexion, extension, rotation, and elbow flexion and extension.

The Hill model describes muscle as a system of three elements, providing a foundational framework for understanding muscle contraction dynamics [18]. This model is essential for creating musculoskeletal simulations in OpenSim, which enables the analysis of upper limb movements, including shoulder and elbow joint functions. Using the Hill model, OpenSim can simulate various motions such as flexion, extension, adduction, and rotation, enhancing the study of human biomechanics [19,20,21,22].

**Figure 4:**
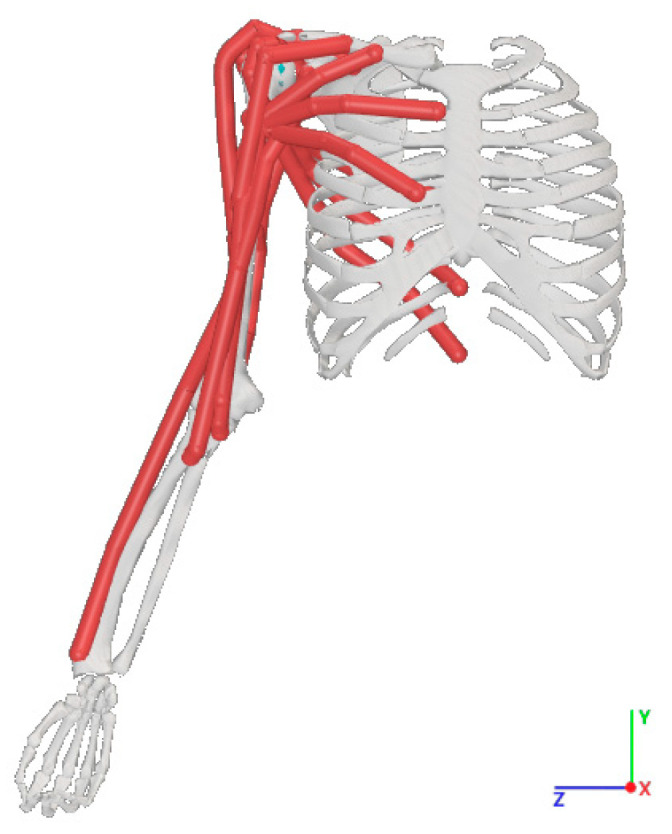
Musculoskeletal model in OpenSim.

This study’s model is based on Saul et al.’s research in OpenSim and includes the shoulder, elbow, wrist, and hand joints and muscles, simplified for this study (Figure 4).

In forward dynamics, the acceleration of the coordinates is described based on Newton’s second law, which considers the inertia and the forces applied to the skeletal structure treated as a set of rigid bodies:

(1) $$\ddot{q}={\left[M\left(q\right)\right]}^{-1}\left\{\tau +C\left(q,\dot{q}\right)+G\left(q\right)+F\right\}$$

In this equation, $\ddot{q}$ is the acceleration caused by the joint torque τ; $C\left(q,\dot{q}\right)$ represents the Coriolis and centrifugal forces, which are functions of position and velocity; $G\left(q\right)$ is the gravitational force; F is the net force acting on the model; and ${\left[M\left(q\right)\right]}^{-1}$ is the inverse of the mass matrix.

The joint torque τ is the sum of the net torques produced by the relevant muscles:

(2) $$\tau =\sum \tau_{m}$$

The net torque $\tau_{m}$ generated by a single muscle force can be expressed as

(3) $$\tau_{m}=\left[R\left(q\right)\right]\cdot f\left(a,l,\dot{l}\right)$$

The net muscle torque $\tau_{m}$ is the result of the moment arm $R\left(q\right)$ multiplied by the muscle force $f\left(a,l,\dot{l}\right)$, where the muscle force $f\left(a,l,\dot{l}\right)$ is a function of muscle activation a, muscle fiber length l, and muscle fiber contraction velocity $\dot{l}$. The muscle fiber contraction velocity $\dot{l}$ can be expressed as

(4) $$\dot{l}=\Lambda \left(a,l,q,\dot{q}\right)$$

(5) $$\dot{a}=A\left(a,x\right)$$

**Figure 5:**
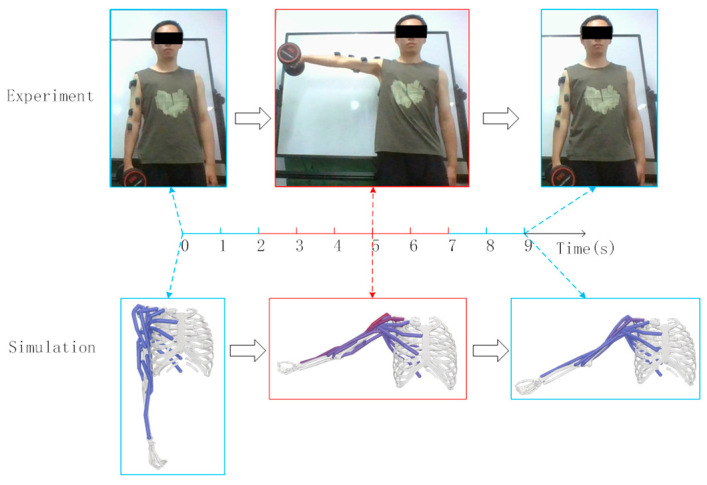
Experimental and simulation of shoulder joint abduction and adduction.

**Figure 6:**
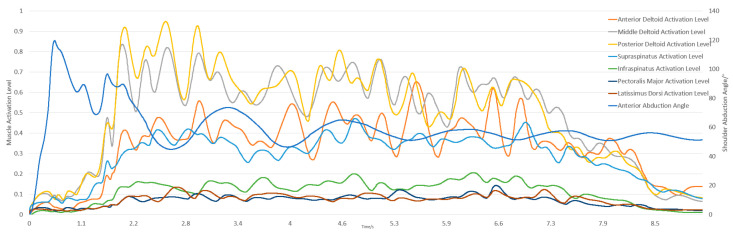
Simulation results of shoulder joint abduction and adduction.

The simulation integrates dynamic equations from the initial state using a 5th-order Runge–Kutta–Fehlberg integrator to calculate muscle activation and contraction velocities. This activation is based on abduction and adduction movements with a 5 kg dumbbell, starting from an upright position and moving to 90 degrees. The experimental and simulation conditions are shown in Figure 5 and Figure 6.

**Figure 7:**
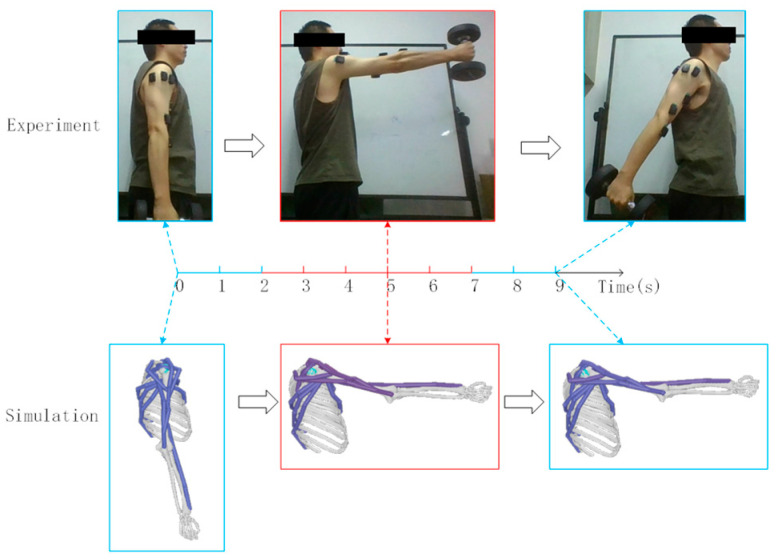
Experimental and simulation of shoulder flexion and extension.

**Figure 8:**
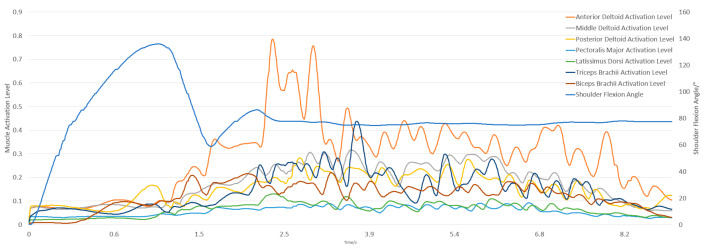
Simulation results of shoulder joint flexion and extension.

The subject held a 5 kg dumbbell, starting from an upright position for 2 s, flexing to 90 degrees for 5 s, and returning to the upright position for another 2 s, totaling 9 s. The model’s initial position in OpenSim matched the experiment, with shoulder elevation, elbow flexion at 0°, and shoulder rotation at 90°. Figure 7 and Figure 8 illustrate the experimental and simulation movements, joint angles, and muscle activation outputs.

**Figure 9:**
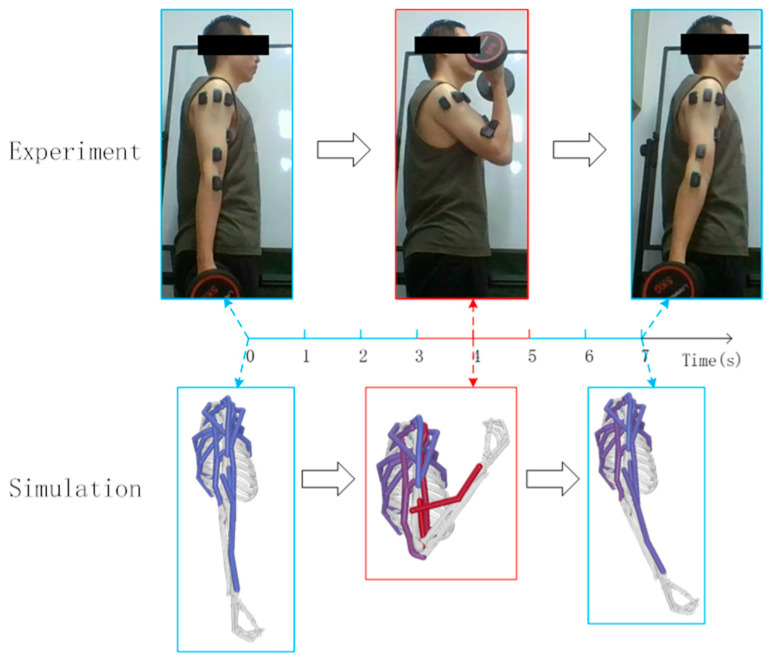
Experimental and simulation of elbow flexion and extension.

**Figure 10:**
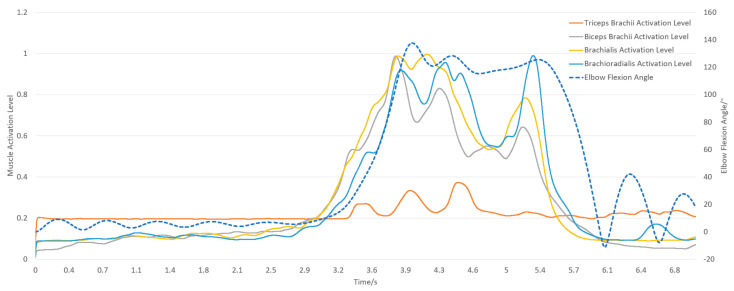
Simulation results of elbow flexion and extension.

The subject held a 5 kg dumbbell, starting upright for 3 s and then flexing the forearm to 130° for 2 s before returning to the upright position for another 2 s, totaling 7 s. In OpenSim, the initial position matched the experiment, with shoulder elevation at 10° and elbow flexion at 0°. Figure 9 and Figure 10 show the experimental and simulation movements, including joint angles and muscle activation outputs.

In summary, based on the simulation results, the musculoskeletal model established in this section, consisting of two joints and eleven muscles, is essentially capable of performing muscle contraction-driven shoulder abduction and adduction in the coronal plane; flexion and extension in the sagittal plane; and elbow flexion and extension.

### 3.2. Data Collection and Preprocessing

**Figure 11:**
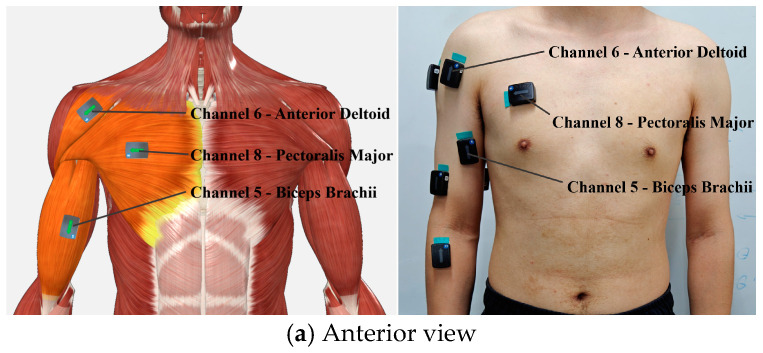

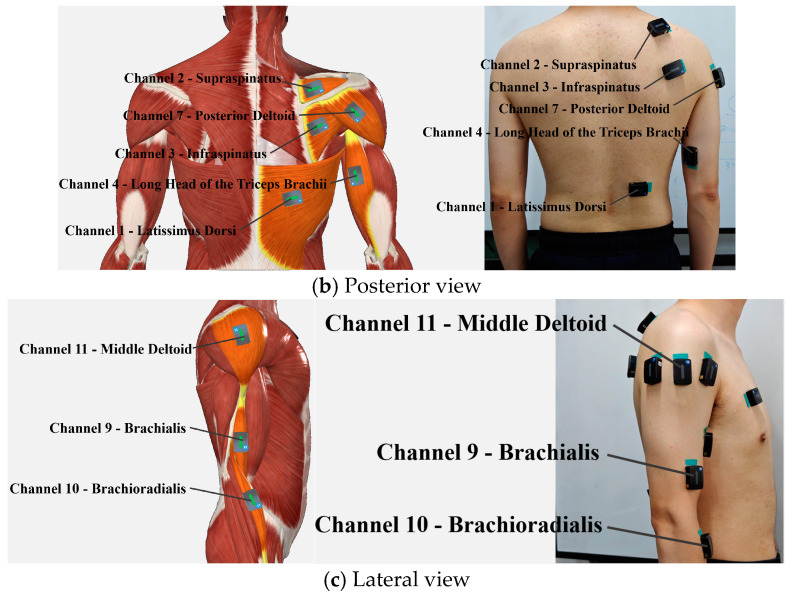
Sensor placement diagram.

This study used Delsys^®^ Trigno sEMG sensors(Delsys Inc., Massachusetts, USA), transmitting data wirelessly to a base station at a sampling frequency of 2000 Hz. The base station connects to a PC for real-time data processing. In the preprocessing and noise reduction of sEMG signals, sensor placement was optimized, wireless RF technology was used to reduce motion artifacts, a 4th-order Butterworth band-pass filter (20 Hz to 450 Hz) was applied to remove noise, and a notch filter was used to eliminate 50 Hz power line interference. Finally, rectification and envelope processing were performed to extract clear electromyographic signals. This study primarily focuses on the movement of the shoulder and elbow joints of the upper limb. Based on anatomical knowledge, the muscle functions related to the shoulder and elbow joint movements, and the measurability of sEMG signals, the main muscles responsible for driving the movement of these two joints were selected for signal collection. Muscles such as the extensor digitorum, extensor carpi radialis brevis, and palmaris longus muscle were excluded. This study focuses on shoulder and elbow movements, selecting eleven key muscles for data collection: latissimus dorsi, supraspinatus, infraspinatus, triceps brachii, biceps brachii, anterior deltoid, posterior deltoid, pectoralis major, brachialis, brachioradialis, and middle deltoid. The sensor placement is shown in Figure 11.

**Figure 12:**
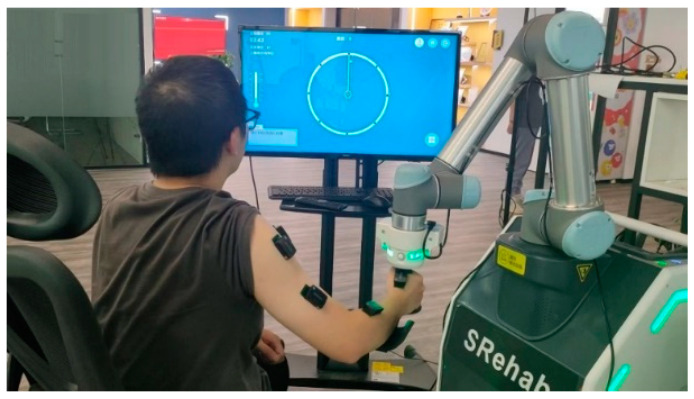
Data acquisition.

First, data collection for the isometric contraction experiment was conducted. After placing the sensors, as shown in Figure 11, subjects sat about 0.3 m in front of the robot, with their right hand grasping the end effector and forearm resting on the support plate. They then performed movements prompted by the interface (Figure 12). The robot restricted movement to a horizontal area of 0.6 m × 0.3 m and applied a maximum resistance of 25 N against the movement. Subjects were instructed to move smoothly to simulate isotonic contraction.

**Figure 13:**
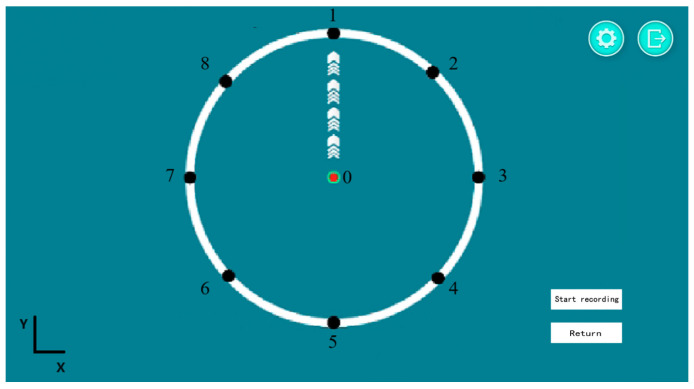
Screenshot of the interface.

The position of the robot’s end effector was indicated on the screen as a red circular cursor (Figure 13). Movements started at the center point (0) and followed directional cues to black circular points (1–8) arranged clockwise. Returning from point (8) to the center completed one set. Each subject performed five sets, with 1–2 min of rest between sets. During each set, the end effector’s position coordinates (*x*, *y*) and six-dimensional force measurements (*Fx*, *Fy*) were recorded at 60 Hz, while sEMG data were collected at 2000 Hz. Raw data were accessible via the data analysis interface.

**Figure 14:**
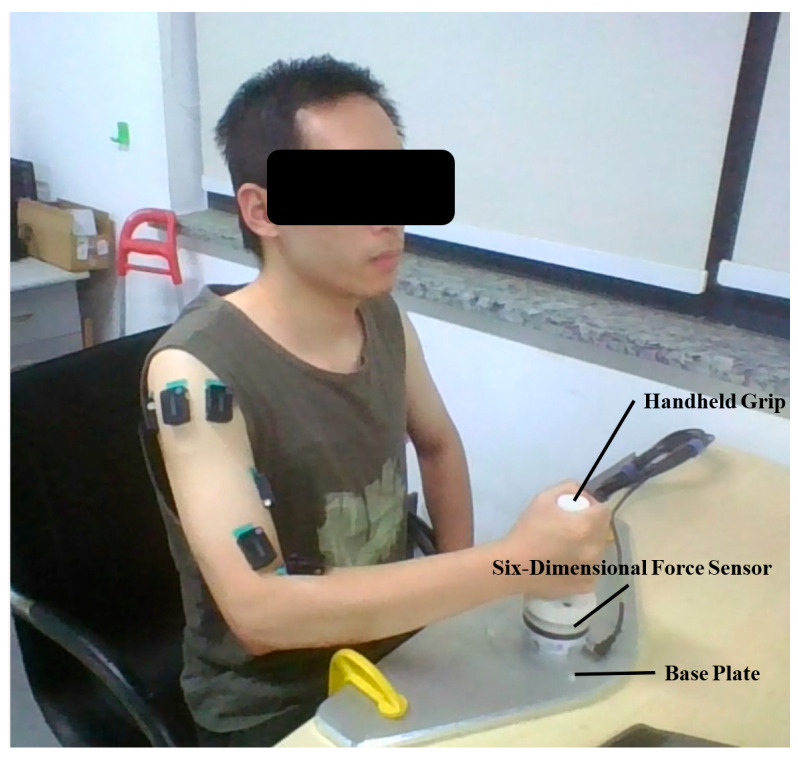
Isometric contraction experiment.

Next, data collection for the isometric contraction experiment was conducted. Figure 14 shows the equipment and setup for collecting isometric contraction data. The hardware system mainly consists of a handheld grip, a six-dimensional force sensor, and a base plate, among other components.

**Figure 15:**
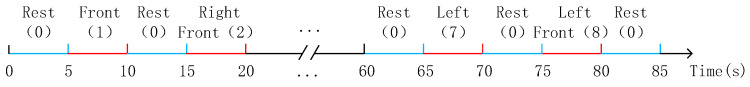
Muscle contraction sequence.

**Figure 16:**
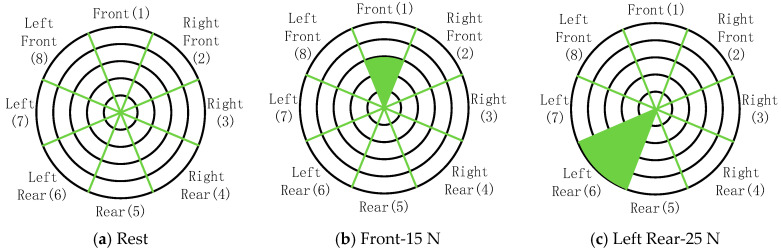
Examples of isometric contraction settings.

During data collection, subjects placed the sensors, as shown in Figure 11, and sat in front of a table, approximately 0.2 m from the grip. They performed isometric contractions in eight directions (coded 1 to 8), with rest intervals (coded 0) in between. Each movement sequence followed a predefined pattern (Figure 15 and Figure 16) and lasted 85 s, with rest and contraction phases at 5 s each. Seven subjects completed five sets of actions at force levels of 5 N, 10 N, 15 N, 20 N, and 25 N, resting for 1 min between sets.

Finally, data collection was collected for the maximum voluntary contraction of the eleven muscles used in this study. Subjects were required to perform designated movements or contract their muscles with maximum force at specified joint angles and positions, maintaining this for 3 to 5 s. To ensure the consistency and reliability of the results, each action was repeated five times, with a rest interval of 1 to 2 min between each repetition to avoid the effects of fatigue accumulation. Normalizing the sEMG signal amplitude to the signal level in MVC allows the force level between individuals to be compared more fairly, and the influence of individual differences can be reduced.

**Figure 17:**
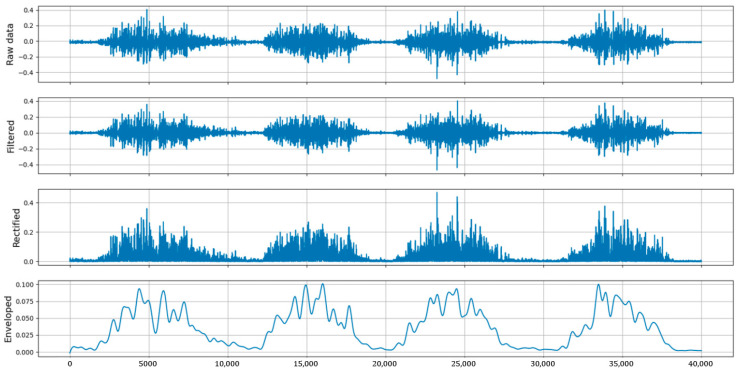
Comparison between raw data and preprocessed data.

During sEMG signal collection, various noise sources can negatively impact signal quality. Optimizing sensor placement is essential to reduce crosstalk from ECG and unrelated muscle activity. Additionally, wireless data transmission can minimize motion artifacts caused by cables. While these measures improve signal quality, further filtering is necessary to extract reliable sEMG signals. The most valuable information is found between 20 Hz and 500 Hz, so band-pass filtering is applied. This study uses a fourth-order Butterworth filter from 20 Hz to 450 Hz and a notch filter to eliminate 50 Hz power line interference [23,24,25]. Processed sEMG signals undergo rectification and envelope detection to assess muscle activation [26]. Figure 17 shows the raw sEMG data, band-pass filtered data, rectified data, and the envelope.

### 3.3. Structure and Training of the Classification Model

**Figure 18:**
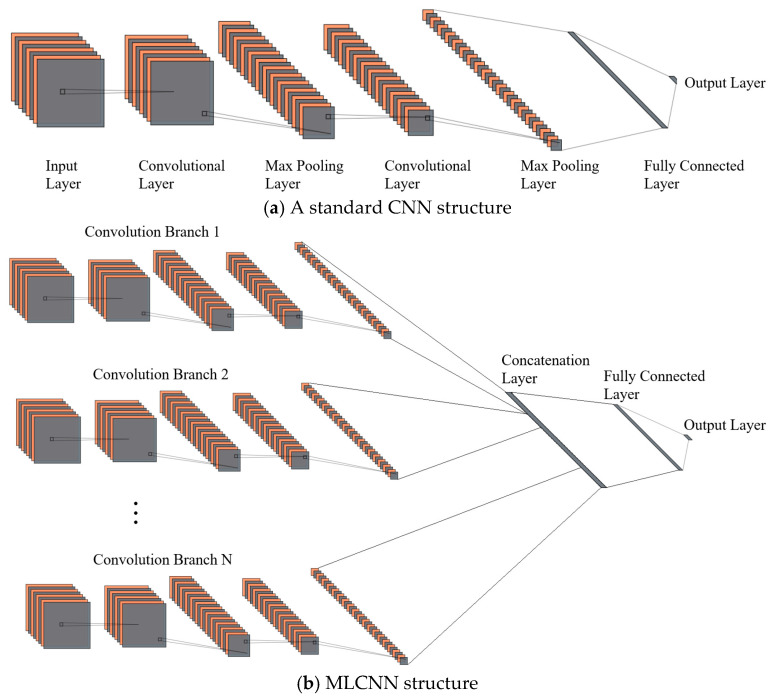
Common CNN and MLCNN structure.

A CNN consists of critical components: convolutional layers, activation functions, pooling layers, and fully connected layers (Figure 18a). An MLCNN is a specialized architecture that integrates multi-stream convolution operations with large pooling windows to enhance performance by parallel processing features at different scales. This approach improves robustness in capturing signal features, allowing the model to retain critical information even if some features are fragmented. Its structure is shown in Figure 18b.

**Figure 19:**
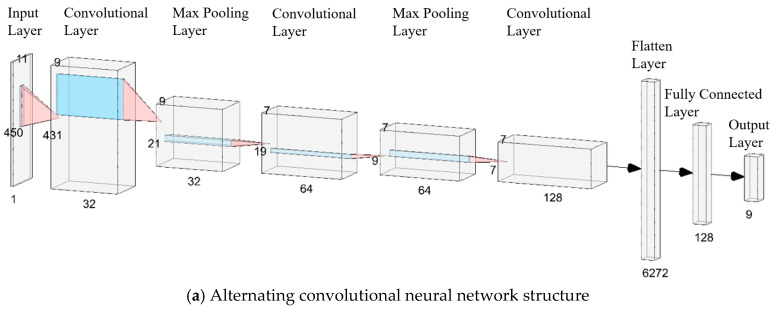

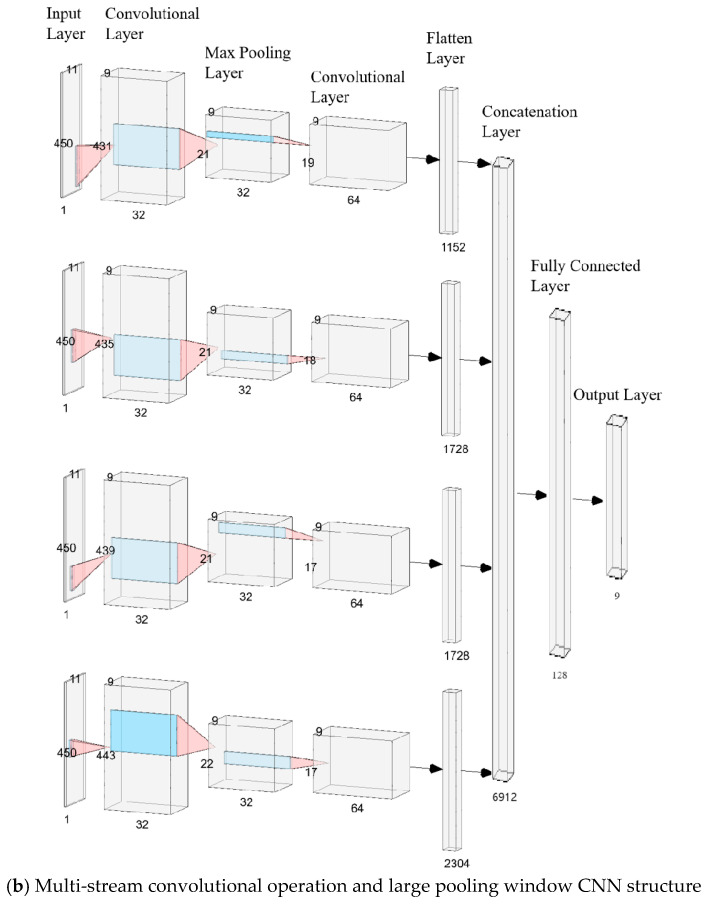
The CNN and MLCNN structures in this study.

Based on the deep learning framework Keras in Python, an alternating convolutional CNN and MLCNN are constructed, as shown in Figure 19a,b, respectively [27]. The numbers in the figures represent the dimensions of each tensor.

**Figure 20:**
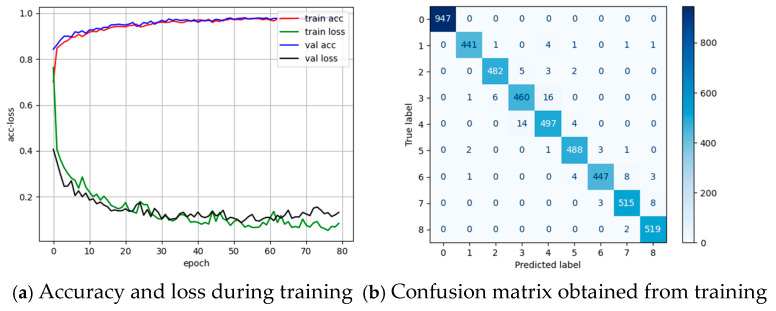
Accuracy and loss of the training and confusion matrix.

Based on data from 105 isometric contraction experiments with Subject 2, the data are divided into seven groups by experiment dates. The original data are segmented into 450-point segments for model input. For each training session, six groups are selected, with 80% as the training set and 20% as the validation set. The seventh group serves as an external cross-validation set. Figure 20a shows the accuracy and loss during training, while Figure 20b presents the confusion matrix. The model performs well, with most data classified correctly. Confusion primarily arises between Category 3 (force applied to the right) and Category 4 (force applied to the right and backward) due to the proximity of muscle force application sites for these movements.

**Figure 21:**
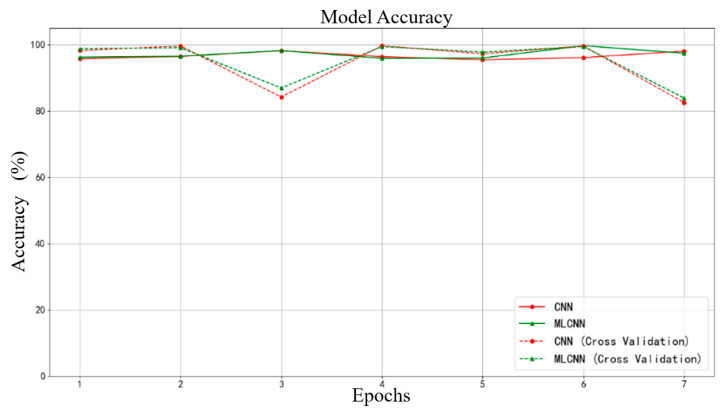
Accuracy of alternating convolutional CNN and MLCNN in cross-validation.

The results of the seven training sessions and cross-validation are shown in Figure 21. Among the models obtained from the seven training sessions, the average accuracy of the CNN model is 96.62%, with an average accuracy of 94.44% on the cross-validation set. The average accuracy of the MLCNN model is 97.13%, with an average accuracy of 95.04% on the cross-validation set.

**Figure 22:**
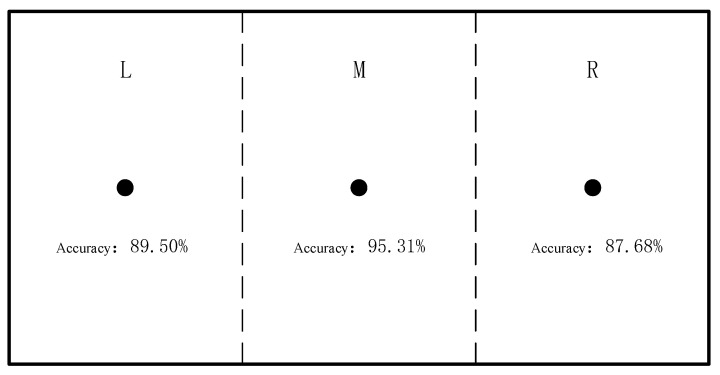
Accuracy of MLCNN models in different areas.

In summary, the performance of the MLCNN model is superior to that of the CNN model. Therefore, based on the experimental space and data division, the MLCNN models trained in regions L, M, and R are obtained, and the accuracy of these models is shown in Figure 22.

Optuna, proposed by Takuya Akiba et al. in 2019, is an automated hyperparameter optimization framework specifically designed for machine learning tasks [28]. It is widely used in tuning hyperparameters for both deep learning and traditional machine learning models, effectively exploring and determining near-optimal model configurations. Additionally, it improves optimization efficiency through pruning techniques, reducing the waste of computational resources. Optuna also provides visualization tools for the hyperparameter tuning process, making it easier for users to understand and analyze the optimization workflow.

In Optuna, users need to define an objective function, which is typically the model’s loss function or accuracy metric. Through multiple trials in a study, Optuna searches for the hyperparameter combinations that optimize the objective function, i.e., minimizing the loss function or maximizing accuracy. Using Optuna, the existing MLCNN model is tuned by setting the return value of the objective function as the accuracy, making the goal of optimization to maximize accuracy. A total of 50 optimization trials are conducted. The seven hyperparameters and their optimization ranges are shown in Table 1.

## 4. Research on Interactive Control of Rehabilitation Robot Based on Motion Intention

### 4.1. Kinematics of iReMo^®^

In robotics kinematics, the velocity describes the relationship between the linear and angular velocities of the end effector and the joint velocities. This relationship is represented by the Jacobian matrix in forward kinematics. The Jacobian matrix can generally be derived using either analytical or geometric methods [29]. The analytical Jacobian matrix $J_{a}(q)$ is derived directly by differentiating the forward kinematics, while the geometric Jacobian matrix $J_{g}(q)$ is obtained from geometric properties, where each joint velocity is related to the changes in the linear and angular velocities of the end effector. The relationship between these two representations can be expressed as

(6) $$J_{a}\left(q\right)=\left[\begin{array}{cc} I & 0\\ 0 & B^{-1}\left(\alpha \right) \end{array}\right]J_{g}\left(q\right)$$

Here, the Euler angles α=[φ,θ,ψ], $B\left(\alpha \right)=\left[\begin{array}{ccc} cos\psi sin\theta & -sin\psi & 0\\ sin\psi sin\theta & cos\psi & 0\\ cos\theta & 0 & 1 \end{array}\right]$. Assuming that $B\left(\alpha \right)$ is a non-singular matrix, this chapter primarily focuses on the velocity relationship between the workspace and the joint space. Therefore, the geometric Jacobian matrix $J_{g}(q)$ will be used for the subsequent derivations.

The geometric Jacobian matrix can be divided into a linear velocity part and an angular velocity part [30,31]. These parts, $J_{v,i}$ (linear velocity part) and $J_{\omega ,i}$ (angular velocity part), are determined by whether the joint is a rotational joint or a prismatic joint.

(7) $$J_{i}=\left[\begin{array}{c} J_{v,i}\\ J_{\omega ,i} \end{array}\right]=\left\{\begin{array}{c} \left[\begin{array}{c} z_{i-1}^{0}\times \left(o_{n}^{0}-o_{i-1}^{0}\right)\\ z_{i-1} \end{array}\right],\;Rotational\;joint\\ \left[\begin{array}{c} z_{i-1}\\ 0 \end{array}\right],Prismatic\;joint \end{array}\right.$$

The robot consists of six rotational joints, so its Jacobian matrix can be expressed as

(8) $$J_{i}=\left[\begin{array}{c} J_{v,i}\\ J_{\omega ,i} \end{array}\right]=\left[\begin{array}{c} z_{i-1}^{0}\times \left(o_{n}^{0}-o_{i-1}^{0}\right)\\ z_{i-1} \end{array}\right],\;i\in \left\{1,\;2,\;3,\;4,\;5,\;6\right\}$$

The Jacobian matrix of the robotic arm relates the joint velocity vector to the end-effector velocity vector $\xi ={\left[v^{T},\omega^{T}\right]}^{T}$. The skew-symmetric matrix S(ω(t)) can be used to determine the angular velocity vector $\omega_{n}^{0}$ in the Jacobian matrix of the robotic arm. The definition of the skew-symmetric matrix S(ω(t)) is as follows:

(9) $$S\left(\omega_{n}^{0}\right)={\dot{R}}_{n}^{0}{\left(R_{n}^{0}\right)}^{T}=\left[\begin{array}{ccc} 0 & {-\omega }_{n,z}^{0} & \omega_{n,y}^{0}\\ \omega_{n,z}^{0} & 0 & -\omega_{n,x}^{0}\\ {-\omega }_{n,y}^{0} & \omega_{n,x}^{0} & 0 \end{array}\right]$$

The linear velocity of the end effector can be directly obtained from the following equation:

(10) $$v_{n}^{0}={\dot{o}}_{n}^{0}$$

Therefore, the Jacobian matrix $J\epsilon R^{6\times n}$ of the robotic arm can be defined separately by the linear velocity part and the angular velocity part:

(11) $$v_{n}^{0}=J_{v}\dot{q}$$

(12) $$\omega_{n}^{0}=J_{\omega }\dot{q}$$

where $J_{v},\;J_{\omega }\epsilon R^{3\times n}$. Finally, the Jacobian matrix can be expressed as

(13) $$\xi =J\dot{q}$$

where $\xi =\left[\begin{array}{c} v_{n}^{0}\\ \omega_{n}^{0} \end{array}\right]\;$ and $J=\left[\begin{array}{c} J_{v}\\ J_{\omega } \end{array}\right]$.

In iReMo^®^, the robotic arm is controlled via joint velocity inputs; therefore, it is necessary to solve for the velocities in inverse kinematics. Assuming that the Jacobian matrix J is non-singular, it is evident that in the robotic arm studied, J is a square matrix. Thus, the joint velocities of the robotic arm can be solved using the inverse Jacobian matrix obtained through LU decomposition:

(14) $$\dot{q}=J^{-1}\xi$$

From this, the joint velocities of the robotic arm can be solved and controlled based on the desired Cartesian velocity of the end effector in the workspace.

### 4.2. Control System and Implementation Methods

**Figure 23:**
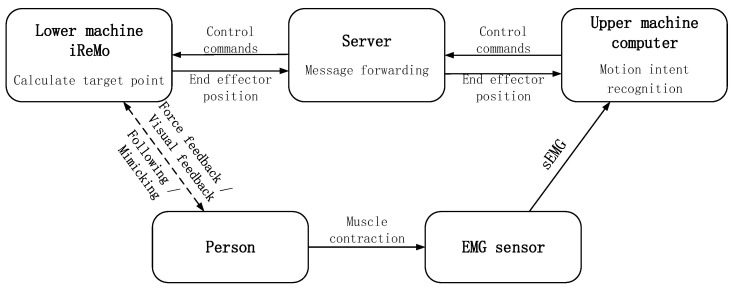
Schematic diagram of system control.

The system comprises the iReMo^®^ lower computer, a server, a host computer, and sEMG sensors, as illustrated in Figure 23. When the user performs active movements, muscle contractions generate EMG signals, which are captured by the sensors and sent to the host computer. The host computer uses the MLCNN model to classify these signals and determine the user’s movement intention. It then generates control commands for the iReMo^®^ end effector to respond accordingly. Both the host computer and iReMo^®^ are connected to the same local area network, using the server for message forwarding to ensure real-time synchronization of commands and status information.

The system features open-loop and closed-loop training based on isometric and isotonic exercises. In isometric training, patients control iReMo^®^ using sEMG signals while holding the handle fixed, allowing the end effector to mimic their movements. In isotonic training, patients hold the end effector directly, creating a closed-loop system with interaction forces. The host computer recognizes nine motion intentions (0 for resting, 1–8 for directional movements). If resting, iReMo^®^ generates no virtual force; otherwise, it calculates a target point (30 mm offset) and produces a 4 N virtual force. This force is converted into desired Cartesian velocity using a PD controller, and joint velocities are computed with the inverse Jacobian matrix, enabling the end effector to mimic the patient’s movements. To avoid vibrations of the robot caused by abrupt changes in virtual force when the motion intention changes, the virtual force is smoothed during transitions to ensure that the movement of iReMo^®^ is as smooth as possible. The specific method for smoothing the virtual force is as follows:

(15) $$F_{smooth}=F_{last}\times \frac{T_{remaining}}{T}+F_{next}\times \frac{T_{passed}}{T}$$

Here, $F_{smooth}$ is the smoothed virtual interaction force, while $F_{last}$ and $F_{next}$ represent the virtual interaction forces before and after the change in motion intention, respectively. T is the smoothing duration, which is set to 0.4 s in this study. $T_{remaining}$ and $T_{passed}$ represent the remaining time and the elapsed time during the smoothing period T; therefore, the relation $T_{remaining}+T_{passed}=T$ is established.

In isotonic training, if the motion intention is resting, no virtual force is generated; if it is active, a virtual interaction force of 6.5 N is directed toward the target point. Actual interaction forces between the hand and the end effector may arise due to the control method not adapting the end effector’s speed based on the sEMG signal amplitude, which can create forces when movement speeds differ with an angle of less than 22.5° between them. Additionally, occasional model misidentification can result in inconsistent movement directions, leading to further interaction forces. The resultant interaction force is formed by combining the actual and virtual forces from sEMG, which is then converted into the desired Cartesian velocity using a PD controller. The inverse Jacobian matrix is used to calculate the joint velocities for execution, achieving closed-loop control. These methods are implemented in the iReMo^®^ controller using C++. The following pseudocode (Algorithm 1) illustrates the control method and PID controller implementation, where the proportional gain Kp is set to 0.15, the integral coefficient Ki is set to 0, and the derivative coefficient Kd is set to 0.5.

**Algorithm 1:** Pseudo code for iReMo^®^ joint speed control. iReMo^®^ Speed ControlInput: F_end_effector: The three-dimensional resultant force applied to the end effector    T_end_effector: The three-dimensional resultant torque applied to the end effector    Q_position_current: The current position of each joint1while(running){2// 3D Force Velocity Control3F_measured = gravity_compensation(Q_position_current, F_end_effector);4F_error = F_reference − F_measured;5F_integrator = F_integrator + F_error;6F_derivative = F_error − F_previous_error;7u_F = Kp * F_error + Ki * F_integrator + Kd * F_derivative;8
9// Angular velocity control of 3D torque10T_measured = T_patient;11T_error = T_reference − T_measured;12T_integrator = T_integrator + T_error;13T_derivative = T_error − T_previous_error;14u_T = Kp * T_error + Ki * T_integrator + Kd * T_derivative;15
16vw[6] = [u_Fx, u_Fy, u_Fz, u_Tx, u_Ty, u_Tz];17Q_velocity_desired = inverse_Jacobian(Q_position_current, vw);18
19UR5.send_speed_command(Q_velocity_desired);20}

**Figure 24:**
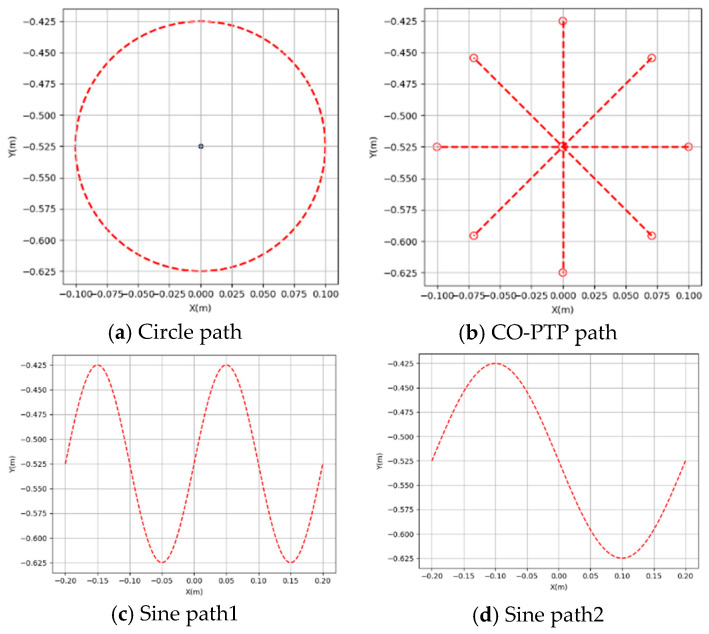
Four standard test paths.

To comprehensively test the system’s motion functionality, four target paths of varying complexity were designed on a horizontal plane measuring 60 cm × 30 cm: a circular path, a CO-PTP path, and two sine paths with different curvatures. The center of the circular path is located at the center of the motion area, with a radius of 0.1 m. The center of the CO-PTP path coincides with the center of the circular path, and its outer points are evenly distributed along the circular path. Sine Path 1 is defined as $y=0.1\sin\left(10\pi x\right)-0.525$ for x∈[−0.2,0.2], while Sine Path 2 is defined as $y=0.1\sin\left(5\pi x\right)-0.525$ for x∈[−0.2,0.2], as shown in Figure 24.

**Figure 25:**
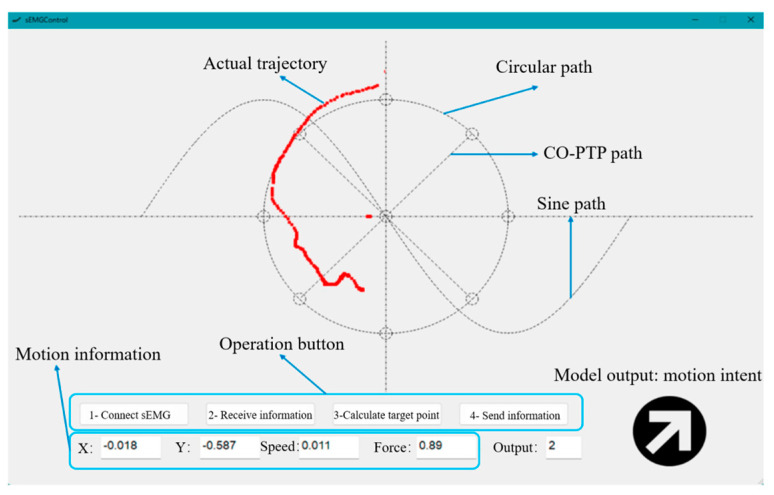
Experimental interface and description.

During the experiment, the placement of the EMG sensors was consistent with the data collection process, with 11 channels of sensors attached to the corresponding muscles, while the upper body remained upright and still. The subjects followed the paths displayed on the testing software interface one by one, receiving feedback through the real-time position displayed on the interface and actively correcting any deviations in their movements, as shown in Figure 25.

To assess the smoothness of movement, two indicators are used:

(1) Smoothness ($\lambda_{s}$): This metric is used to describe the coherence of movement. The calculation method is as follows:

(16) $$\lambda_{s}=\frac{v_{a}}{v_{max}}$$

Here, $v_{a}$ represents the average speed during a segment of movement, while $v_{max}$ is the maximum speed within the same segment. Therefore, the smoothness value ranges from 0 to 1, where a higher value closer to 1 indicates a greater capability demonstrated by the subject during the movement.

(2) Range Deviation ($\lambda_{d}$): This metric is used to describe the positional deviation from the standard trajectory during movement. The calculation method is as follows:

(17) $$\lambda_{d}=\frac{1}{1+R_{x}}$$

(18) $$R_{x}=\frac{1}{L}\cdot \sqrt{\frac{1}{N-1}\cdot \sum_{k=1}^{N}{\left(∆s_{k}\right)}^{2}}$$

Here, *L* represents the actual length of the standard trajectory, and $∆s_{k}$ is the distance from the end effector’s center to the standard trajectory at time k. Therefore, the range deviation value ranges from 0 to 1, where a higher value closer to 1 indicates stronger control abilities demonstrated by the subject during the movement.

To assess the precision of movement, the normalized path length $\lambda_{l}$ is used. This metric represents the ratio of the length of the subject’s actual movement trajectory to the length of the standard trajectory.

(19) $$\lambda_{l}=\frac{L_{r}}{L}$$

Here, $L_{r}$ represents the length of the actual trajectory. When $L_{r}$ is less than the standard trajectory length *L*, the value is less than 1; conversely, when $L_{r}$ is greater than L, the value is greater than l. Thus, the value is generally distributed around 1. However, since the normalized path length cannot describe the similarity between the actual trajectory and the target trajectory, it cannot be used alone to assess movement ability. When the range deviation indicator is close to 1, a normalized path length closer to 1 indicates stronger movement control abilities in the subject.

## 5. Results

### 5.1. Classification Model and Optimization Results

**Figure 26:**
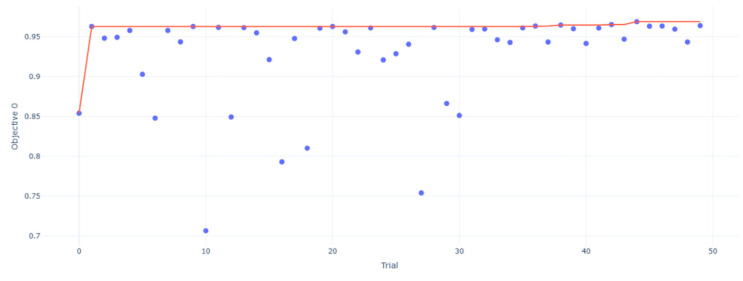
Accuracy of the model M during the optimization process.

**Figure 27:**
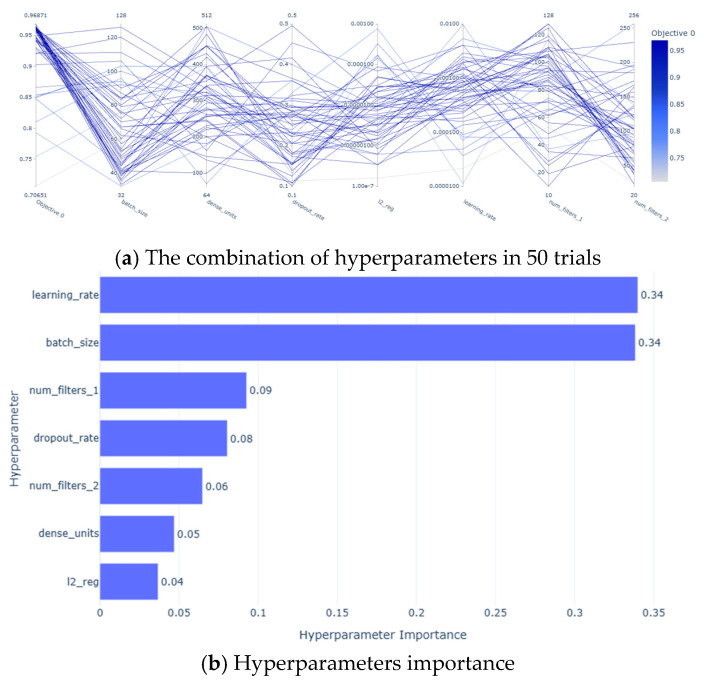
Hyperparameter combinations and their importance.

The information about the subjects is shown in Table 2. The changes in the optimization target accuracy during the model optimization process in region M are shown in Figure 26. In the 50 trials, the accuracy remained above 70%, with the highest accuracy of 96.87% achieved in trial 45. The hyperparameter combinations selected by Optuna during the trials are illustrated in Figure 27a, while the importance of the hyperparameters is depicted in Figure 27b. Among the seven hyperparameters, the learning rate and batch size had the most significant impact on model performance, each with an importance score of 0.34; the combined importance of the other five parameters was 0.32.

**Figure 28:**
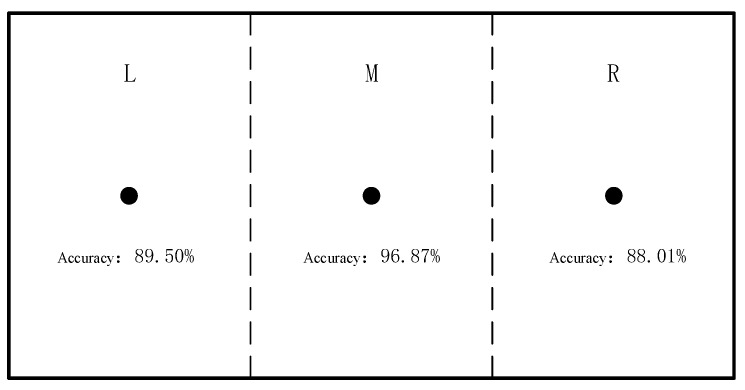
Accuracy of the best model in each area.

For the models in regions L and R, optimization was conducted, and through multiple studies, the optimal parameters for models in the three regions were obtained. The model accuracy under the optimal parameters is shown in Table 3. The model accuracy for regions M and R improved by 1.56% and 0.33%, respectively, compared to before optimization, while the model accuracy for region L was lower than that of the original model. Ultimately, the accuracies of the models for regions L, M, and R were 89.5%, 96.87%, and 88.01%, respectively, as shown in Figure 28. The lower accuracies in regions L and R compared to the central region may be related to the muscle exertion patterns and experimental design.

The classification performance of the isotonic experiments was poor due to unclear muscle contractions and instability, making it unsuitable for classification.

### 5.2. Robot Interaction Control Experiment Results

#### 5.2.1. Comparison of Different Control Methods Under the Same Path

**Figure 29:**
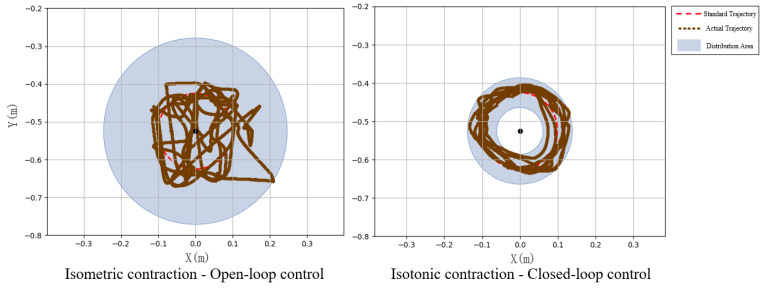
Circular trajectory distribution diagram of closed-loop control and open-loop system.

Subject 2 performed seven experiments on isotonic and isometric contractions, as shown in Figure 29. The red dashed line indicates the standard circular path, while the brown lines depict the experimental trajectories. Under open-loop control, trajectories were irregular and scattered (inner radius: 0.0045 m, outer radius: 0.2460 m). In contrast, closed-loop control yielded more circular and concentrated trajectories (inner radius: 0.0618 m, outer radius: 0.1384 m), demonstrating improved alignment with the standard path.

**Figure 30:**
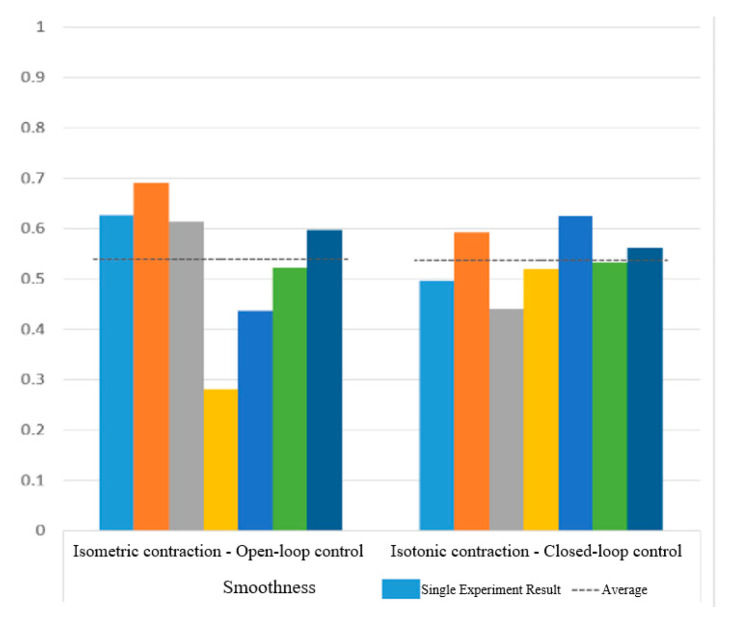
Smoothness of circular path in open-loop and closed-loop system.

**Figure 31:**
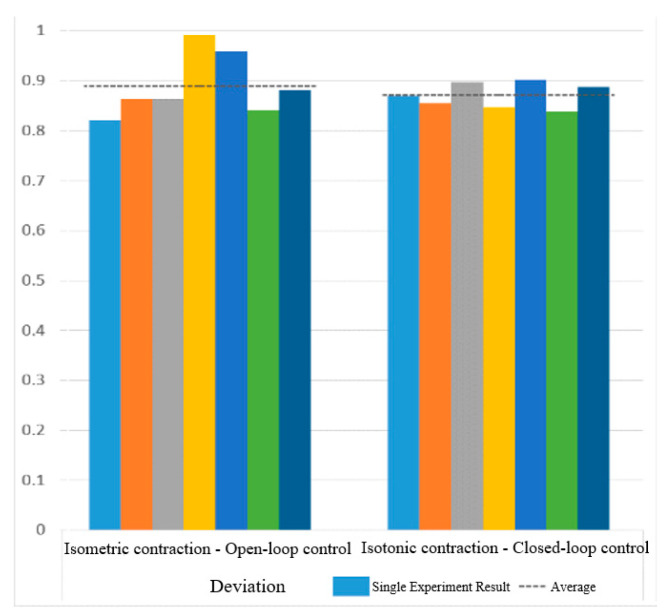
Deviation of circular path in open-loop and closed-loop system.

Figure 30 and Figure 31 show the motion smoothness and range deviation results. Under open-loop control, the average smoothness was 0.5388, with a range deviation of 0.8885. In contrast, closed-loop control had a smoothness of 0.5381 and a range deviation of 0.8711. While both methods had similar smoothness, open-loop control was less stable, exhibiting greater fluctuations than the more consistent closed-loop performance.

**Figure 32:**
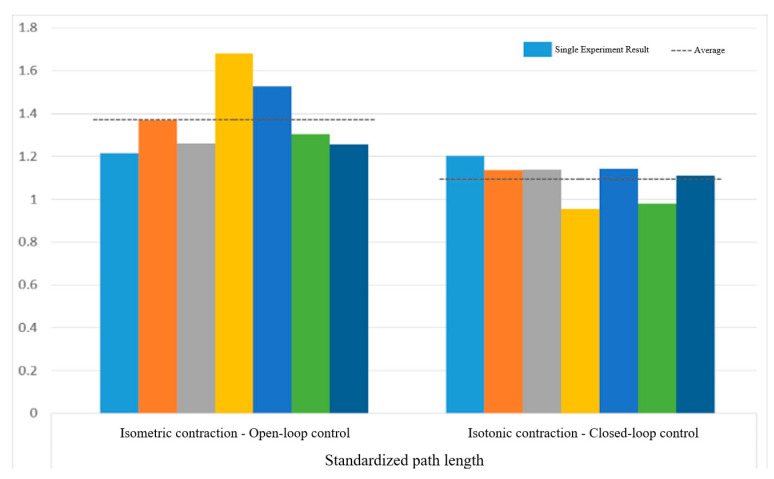
Standardized path length of circular path in open-loop and closed-loop system.

**Figure 33:**
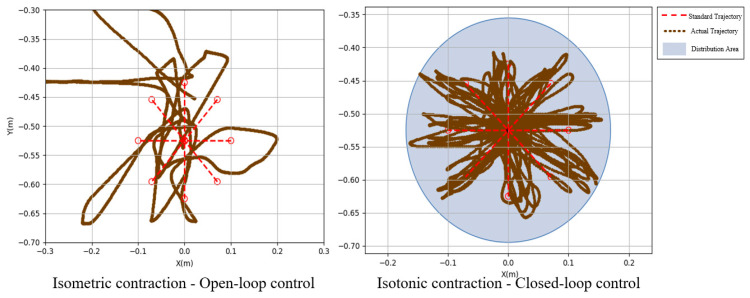
CO-PTP trajectories distribution map.

Figure 32 displays the normalized path length indicators, showing averages of 1.37 for open-loop control and 1.10 for closed-loop control. In Figure 33, the standard CO-PTP path is marked by a red dashed line, while the brown lines represent actual trajectories. Open-loop control exhibited significant divergence from the intended path, complicating the completion of all segments. Conversely, closed-loop control achieved accurate movement but often overshot segment endpoints, indicating challenges in precise stopping.

**Figure 34:**
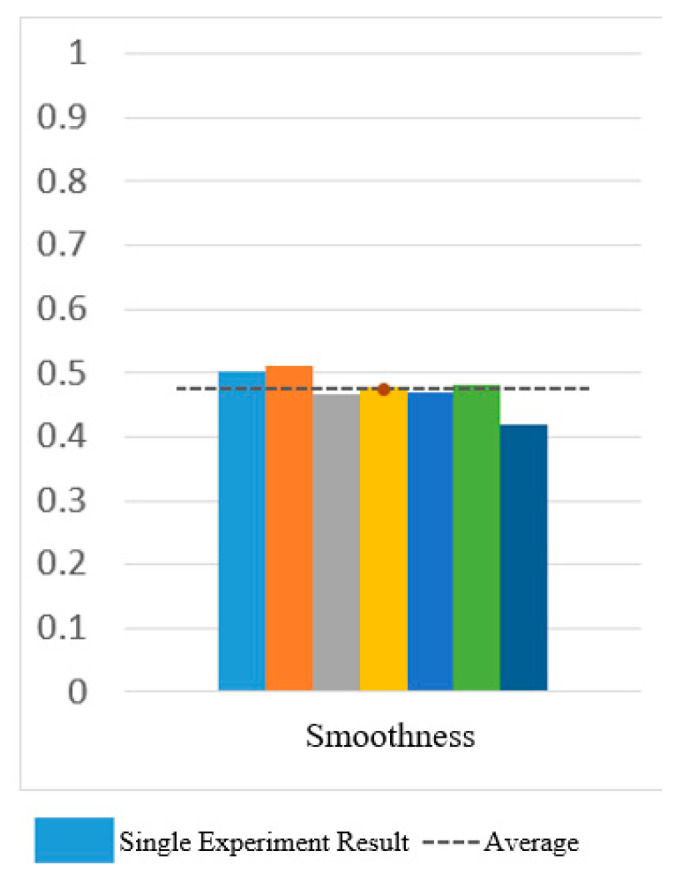
Smoothness of CO-PTP path in closed-loop system.

**Figure 35:**
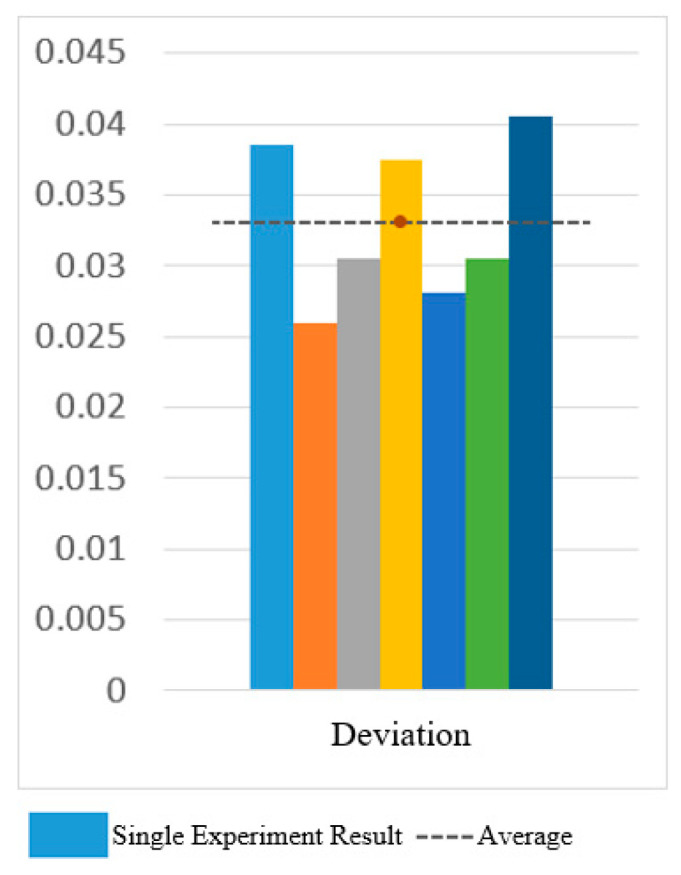
Deviation of CO-PTP in closed-loop system.

**Figure 36:**
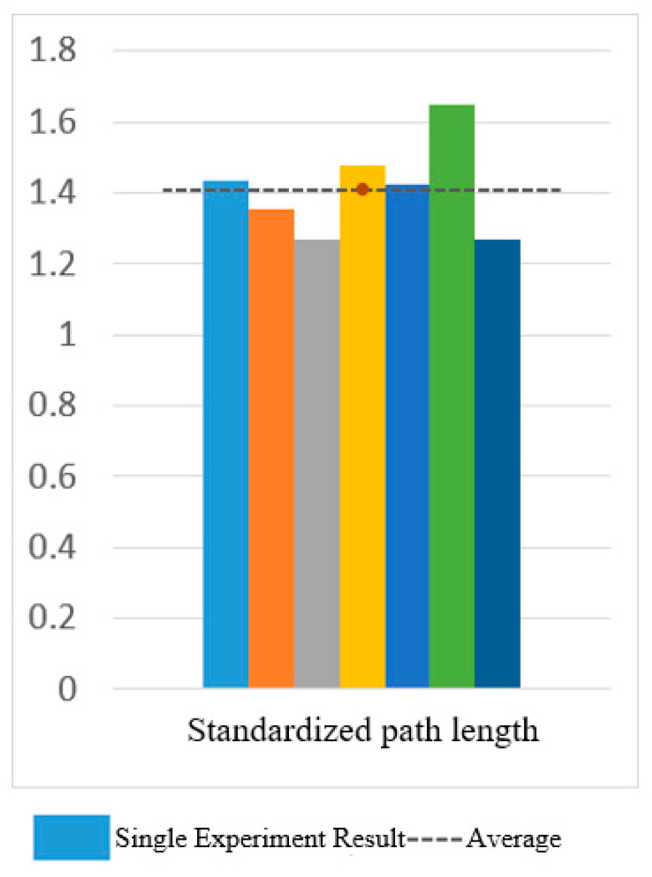
Standardized path length of CO-PTP path in closed-loop system.

Figure 34, Figure 35 and Figure 36 show the results for smoothness, range deviation, and normalized path length in closed-loop control experiments, with averages of 0.48 for smoothness, 0.03 for range deviation, and 1.41 for normalized path length. The consistent smoothness indicates uniformity, while the low range deviation highlights divergence from the standard path during CO-PTP movements.

**Figure 37:**
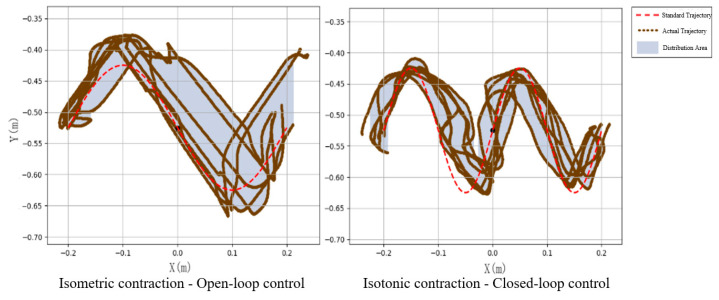
Sinusoidal trajectories distribution map.

Figure 37 illustrates the results for Sinusoidal Path 2 under open-loop control and Sinusoidal Path 1 under closed-loop control. The red dashed line represents the standard path, while the brown lines depict actual trajectories from the seven experiments. Under open-loop control, the trajectories were mostly straight, leading to significant deviations from the standard path. In contrast, closed-loop control generally followed the sine shape but showed discrepancies at the troughs, with none of the experiments fully matching the standard path.

**Figure 38:**
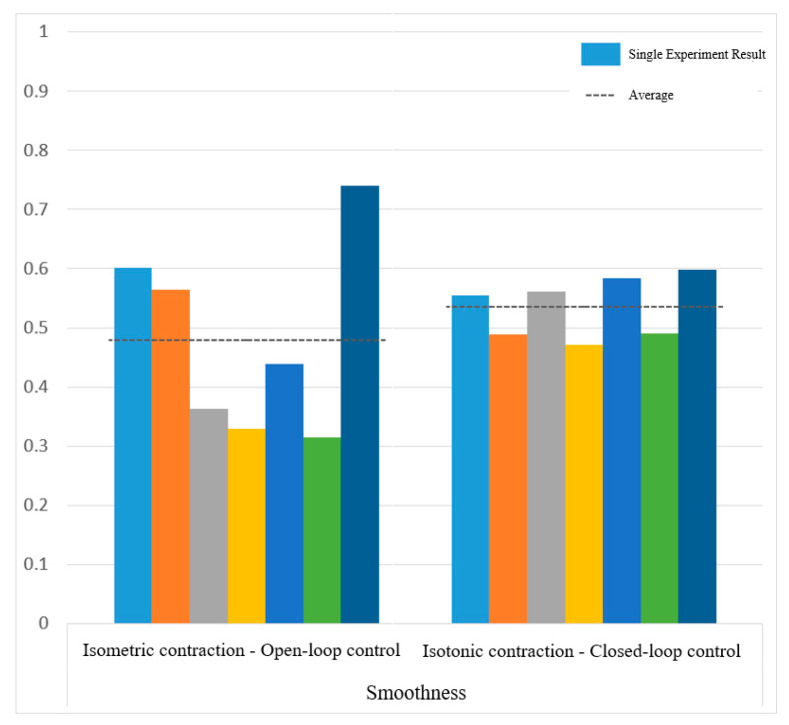
Smoothness of sinusoidal path in open-loop and closed-loop control.

**Figure 39:**
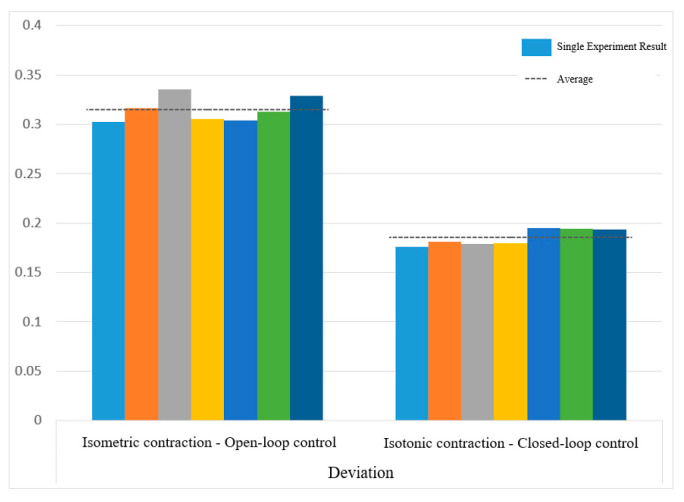
Deviation of sinusoidal path in open-loop and closed-loop control.

**Figure 40:**
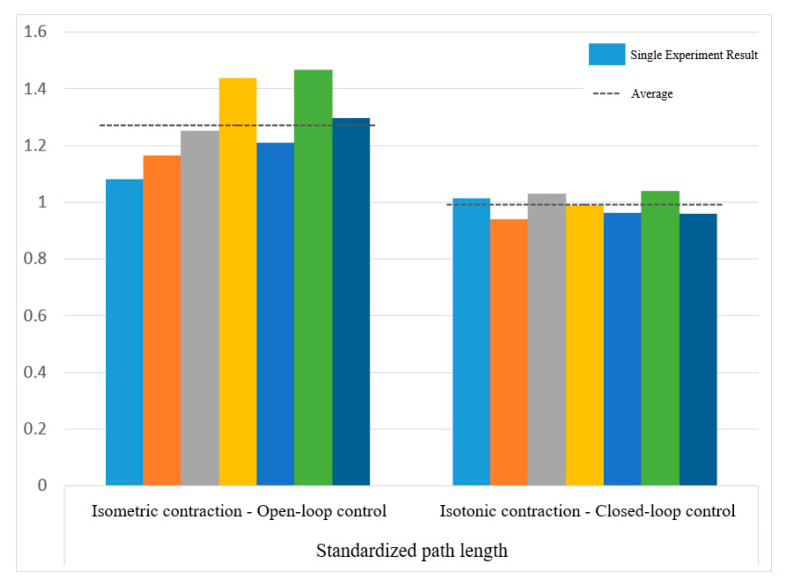
Standardized path length of sinusoidal path in open-loop and closed-loop control.

Figure 38 and Figure 39 show that open-loop control has an average smoothness of 0.48 and a range deviation of 0.32, while closed-loop control averages 0.54 in smoothness and 0.19 in deviation. Thus, motion smoothness is similar, but open-loop control better handles range deviation. Figure 40 presents standardized path length, with open-loop averaging 1.27 and closed-loop at 0.99. Open-loop control exceeds the target length with mostly straight paths, while closed-loop control takes a shorter, more direct route, indicating better trajectory control.

#### 5.2.2. Comparison of Different Paths Under the Same Control Mode

**Figure 41:**
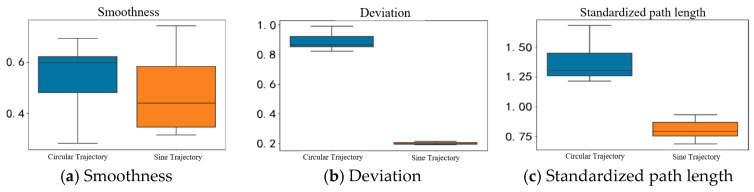
Box plots of two trajectory indicators in open-loop control.

Due to the poor performance of open-loop control under the CO-PTP path, this paper focuses on comparing the circular path and Sine Path 2. In Figure 41, the circular trajectory exhibits a smoother speed variation, with its smoothness distribution concentrated around 1.0, while the sine trajectory is more dispersed. For range deviation, the circular path shows effective error control with values between 0.8 and 1.0, whereas the sine trajectory averages around 0.2, indicating substantial deviations from the target path. Additionally, the circular path length ranges from 1.25 to 1.5, reflecting longer actual movement paths than theoretical lengths, while the sine trajectory ranges from 0.75 to 1.0, suggesting slightly shorter actual paths.

**Figure 42:**
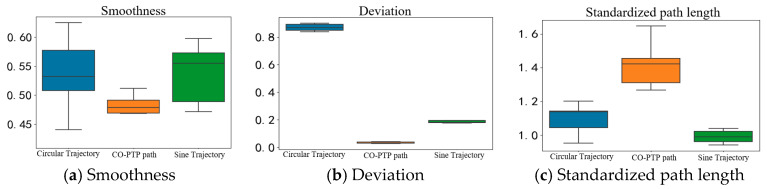
Box plots of three trajectory indicators in close-loop control.

In the closed-loop control experiments, box plots in Figure 42 analyze smoothness, range deviation, and standardized path length for three paths. Motion smoothness centers around 0.5 for all trajectories, indicating a maximum speed approximately twice the average speed. For range deviation, values from 0 to 1 show that higher values indicate smaller deviations from expected trajectories, with the ranking as follows: CO-PTP path > Sine Path 1 > circular path. The CO-PTP path requires the highest control capability due to its straight segments, while the sine trajectory shows lower accuracy at trough positions.

All trajectories exhibit standardized path lengths close to or exceeding 1, indicating that actual movement paths generally exceed the theoretically shortest lengths, aligning with typical upper limb motion patterns in humans.

## 6. Discussion

This study develops an interactive control method based on surface electromyography (sEMG) signals, aimed at providing rehabilitation training for stroke patients with upper limb movement disorders, filling a certain gap in existing technology. The innovation of this study lies in the selection of upper limb muscles based on OpenSim, using sEMG signals for movement intention recognition, and employing the MLCNN model to improve classification accuracy. However, this method still faces numerous challenges and limitations. Compared to existing research in the field, this study has made progress in the classification and recognition of movement intentions and the implementation of control systems, especially by utilizing deep learning models (such as MLCNN) to improve classification accuracy, showing promising results. However, many areas still require improvement. Firstly, due to alterations in muscle electrical activity under varying load and fatigue states, leading to increased non-linearity and instability in the signals, classification accuracy decreases under different force levels and muscle fatigue states, reflecting the model’s inadequate adaptability to complex real-world situations. Secondly, due to limitations in obtaining anatomical parameters, the established musculoskeletal model for the upper limb fails to accurately simulate the dynamic movement of the shoulder joint, leading to discrepancies between the simulation results and the actual conditions. Compared to other studies, such as those that have established complete musculoskeletal models and employed more complex algorithms, this study appears relatively basic, especially in terms of the model’s robustness and adaptability. Other research may demonstrate greater maturity in data processing, signal classification, and model simulation. Therefore, although this study has foundational significance in advancing related fields, its practical applicability and effectiveness require further optimization.

Future research can focus on improving the model’s adaptability and accuracy, particularly in processing sEMG signals under varying muscle force levels and fatigue states. Introducing more experimental data, advanced model optimization techniques, and real-time feedback mechanisms will help enhance the performance of the designed control system, better serving the rehabilitation needs of stroke patients.

## 7. Conclusions

The upper limb rehabilitation robot interactive control method based on surface electromyographic (sEMG) signals developed in this study can effectively support isometric and isotonic training for stroke patients with upper limb motor impairments. Although this study utilizes machine learning and deep learning methods to process sEMG signals and classify movement intentions of the upper limb in the horizontal plane, successfully using electromyographic signals to control the upper limb rehabilitation robot, and achieving isometric and isotonic training for stroke patients with upper limb motor impairments, some shortcomings have been revealed. These include insufficient classification accuracy of the model under different force levels and muscle fatigue states and limitations in the anatomical parameter acquisition of the established musculoskeletal model. Future research should focus on enhancing the robustness of the classification model, optimizing the accuracy of movement intention recognition, and improving the processing of surface electromyographic signals under fatigue conditions. This will lay the foundation for improving rehabilitation outcomes for stroke patients and promote the development of this field.

## Figures and Tables

**Table 1:** Tunable parameters table.

Hyperparameters	Parameter Definition	OMV	Optimization Range
learning_rate	Learning Rate	1 × 10^−3^	[1 × 10^−5^, 1 × 10^−2^]
num_filters_1	Number of Filters in Convolutional Layer 1	32	[10, 128]
num_filters_2	Number of Filters in Convolutional Layer 2	64	[20, 256]
dropout_rate	Dropout Rate	0.5	[0.1, 0.5]
dense_units	Number of Neurons in Fully Connected Layer	128	[64, 512]
batch_size	Batch Size	64	[32, 128]
l2_reg	L2 Regularization Coefficient	None	[1 × 10^−7^, 1 × 10^−3^]

**Table 2:** Subject Information.

Subjects	Age	Gender	Height (cm)	Weight (kg)
1	26	man	172	66
2	28	man	170	60
3	24	man	175	62
4	24	man	176	58
5	24	man	192	80
6	24	man	173	60
7	24	man	185	85

**Table 3:** The hyperparameters and accuracy of the optimized models.

Regions	L	M	R
learning_rate	0.0016036142	0.0007133843	0.0014916109
num_filters_1	113	106	62
num_filters_2	219	44	74
dropout_rate	0.4680741832	0.2770565315	0.4362810779
dense_units	466	261	484
batch_size	75	39	128
l2_reg	1.2438425879	4.7615135451	1.9060718778
accuracy	88.26%	96.87%	88.01%

## Data Availability

The dataset is not publicly available.
